# THE RELATIONSHIP OF PREDEATH GRIEF, BURDEN, AND EMPATHETIC PAIN IN FAMILY CAREGIVERS

**DOI:** 10.1093/geroni/igae098.1514

**Published:** 2024-12-31

**Authors:** Lauren Chrzanowski, Elisabeth McLean, Jonathan Singer

**Affiliations:** Texas Tech University, Lubbock, Texas, United States; Texas Tech University, Lubbock, Texas, United States; Texas Tech University, Lubbock, Texas, United States

## Abstract

Family caregivers may experience pain as an empathetic reaction to their loved ones’ pain – this has not been studied in aging populations. Caregiver distress (such as pre-death grief (PDG)) increases as patient pain worsens. Therefore, this study hypothesized that higher PDG and caregiver burden would result in higher rates of empathetic pain for family caregivers. This study included family members of persons with advanced cancer (N=100) and dementia (N=38). We measured PDG using the PG-12 scale, burden with the Zarit Burden Inventory (ZBI), and used a single item (i.e., How often have you experienced pain (or other symptoms) that the individual with life limiting illness as (e.g., empathetic pains)), dichotomized (0=never; 1=at least once a day-multiple times a day) to measure empathetic pain. Logistic regressions were performed to ascertain the effects of PDG and burden on the likelihood of family caregivers experiencing empathetic pain when controlling for diagnoses and age of care recipient. PDG significantly predicted empathetic pain X2(3, N = 118) = 18.83, p<.001. The model explained 20.4% (Nagelkerke R2) of the variance. Higher PDG score was associated with an increased likelihood of endorsing empathetic pain (OR=.896, 95%CI [1.041, 1.161]). Caregiver burden significantly predicted empathetic pain X2(3, N = 124) =7.81, p=.05. The model explained 8.4% (Nagelkerke R2) of the variance. Higher ZBI score was associated with an increased likelihood of endorsing empathetic pain (OR=.889, 95%CI [1.002, 1.128]). These results provide evidence that higher PDG and burden can lead to downstream effects on the caregiver, including empathetic pain.

